# Exploring the Role of Central Venous Pressure in Cardiac Surgery-Associated Acute Kidney Injury: A Comprehensive Scoping Review

**DOI:** 10.34172/aim.35112

**Published:** 2025-12-01

**Authors:** Maryam Aligholizadeh, Siavash Sangi, Mehrdad Mesbah Kiaei, Mahmoud Reza Mohaghegh, Mohsen Abbasi, Melika Aligholizadeh

**Affiliations:** ^1^Department of Anesthesiology and Operating Room, School of Nursing and Midwifery, Shahid Beheshti University of Medical Sciences, Tehran, Iran; ^2^Department of Anesthesiology and Pain Medicine, School of Medicine, Hasheminejad Kidney Center, Iran University of Medical Science, Tehran, Iran; ^3^Department of Anesthesia, School of Medicine, and Hospital Management Research Center, Health Management Research Institute, Iran University of Medical Sciences, Tehran, Iran; ^4^Department of Anesthesia and Critical Care, Hasheminejad Hospital, School of Medicine, Iran University of Medical Sciences, Tehran, Iran; ^5^Department of Laboratory Sciences, Langroud School of Allied Medical Sciences, Guilan University of Medical Sciences, Langroud, Iran

**Keywords:** Acute kidney injury, Central venous pressure, Coronary artery bypass grafting

## Abstract

**Background:**

Acute kidney injury (AKI) is a critical complication, affecting up to 30% of coronary artery bypass grafting (CABG) patients, and contributing to significant morbidity and mortality. Recent studies indicate that increased central venous pressure (CVP) might significantly contribute to the development of AKI by causing venous congestion and impairing renal blood flow. However, the association between CVP and AKI in patients undergoing CABG has not been thoroughly investigated. This scoping review evaluates the current evidence on CVP as a hemodynamic marker associated with AKI in adults undergoing cardiac surgery with cardiopulmonary bypass (CPB), with a particular focus on CABG where reported.

**Methods:**

This scoping review, conducted over 12 weeks, followed the PRISMA-ScR guidelines and Arksey and O’Malley framework. A systematic search of PubMed, Scopus, Web of Science, and MEDLINE (2016–2024) identified studies on adult CPB-supported cardiac surgery, including CABG. Eligible studies reported quantitative CVP (intra- or postoperative) and standardized AKI criteria. No formal bias assessment was performed; data extraction was independently conducted by two reviewers using a standardized form.

**Results:**

Of 1,717 studies screened, 16 met the inclusion criteria, mostly retrospective cohorts involving CABG patients. Overall, elevated CVP showed a positive association with postoperative AKI, though thresholds varied (intraoperative 6.5–12 mm Hg; postoperative>6.6–10.3 mm Hg). Several studies revealed a synergistic effect between high CVP and low mean arterial pressure (MAP). Despite consistent trends, heterogeneity in design and CVP assessment limits comparability. Most studies used the KDIGO criteria for AKI definition.

**Conclusion:**

High CVP is commonly linked to the occurrence of AKI in patients undergoing cardiac surgery. The evidence mapped in this review suggests a potential role for CVP monitoring in perioperative care, though clinical recommendations require validation through prospective trials. Future research should focus on establishing standardized CVP thresholds and evaluating their utility in AKI risk stratification.

## Introduction

 With global advancements in living standards and rising life expectancy, the prevalence of coronary artery disease (CAD) has increased markedly. Coronary artery bypass grafting (CABG) remains a cornerstone intervention for patients with advanced CAD and other complex cardiovascular conditions, significantly improving survival and quality of life. However, like other major cardiac surgeries involving cardiopulmonary bypass (CPB), including valve replacement and aortic repair, CABG is associated with a substantial risk of postoperative complications.^1,2^ Acute kidney injury (AKI) is among the most common and serious complications following cardiac surgery. According to recent research, the incidence of cardiac surgery-associated AKI (CSA-AKI) varies between 10% and 40%, depending on the diagnostic criteria used. The development of CSA-AKI has been associated with a 3–8-fold increase in mortality, longer durations of intensive care and overall hospital stay, as well as elevated healthcare costs.^3^ Cardiac surgery poses unique challenges, including CPB, aortic cross-clamping, high-volume blood transfusions, and extensive use of vasopressors.^4,5^ These factors disrupt renal perfusion, trigger ischemia-reperfusion cycles, and promote oxidative stress and inflammation, all of which play a role in AKI development. Historically, renal hypoperfusion has been viewed as the dominant hemodynamic mechanism contributing to AKI, leading clinicians to focus primarily on maintaining adequate mean arterial pressure (MAP) and cardiac output. Although a MAP ≥ 65 mm Hg is commonly targeted to reduce AKI risk, recent evidence suggests that perfusion pressure alone may not fully explain postoperative renal dysfunction.^6,7^ Cardiac surgery entails unique insults—including CPB, aortic cross-clamping, massive transfusions, and vasopressor support—that collectively compromise renal perfusion, drive ischemia-reperfusion injury, and amplify oxidative stress and inflammation.^8,9^ Emerging evidence now highlights venous congestion as an equally important yet underrecognized factor in CSA-AKI pathophysiology. Elevated central venous pressure (CVP), an indicator of venous congestion, directly contributes to renal venous hypertension and reduces the renal arteriovenous pressure gradient.^10,11^ This hemodynamic alteration impairs renal blood flow, decreases glomerular filtration rate, and exacerbates intrarenal pressure. Elevated CVP activates both the sympathetic nervous system and the renin–angiotensin–aldosterone system, leading to increased sodium reabsorption, fluid accumulation, and heightened systemic inflammatory responses.^6,12^

**Figure 1:**
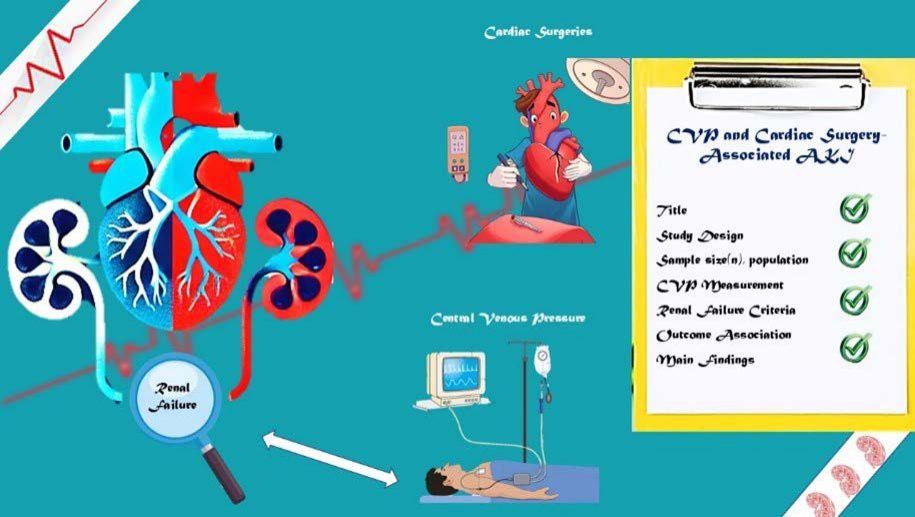
Graphical Abstract: Central venous pressure and cardiac surgery-associated acute kidney injury – a scoping review

 Despite the widespread use of CVP monitoring during and after cardiac surgery, there remains no consensus on the threshold values at which CVP becomes deleterious to renal function in CABG patients.^13,14^ Moreover, the relative impact of intraoperative versus postoperative CVP elevations has not been systematically synthesized, and it is still unclear whether CVP acts as an independent predictor of CSA-AKI or merely reflects other hemodynamic derangements such as right ventricular dysfunction or volume overload.^13,15^ These uncertainties underscore the need for comprehensive evidence mapping in this domain. The rationale for this scoping review is to systematically chart and synthesize the available literature regarding CVP and its association with AKI following CABG and other cardiac surgeries, identifying existing knowledge gaps and research trends. By applying the Population–Concept–Context (PCC) framework, this review focuses on adult patients undergoing cardiac surgery (Population), explores the concept of CVP monitoring as a potential predictor or contributing factor to AKI (Concept), and examines its application and implications across intraoperative and postoperative settings (Context). This scoping review critically examines the available evidence on CVP’s association with AKI in this population, aiming to elucidate its clinical utility, limitations, and potential as a hemodynamic marker. By bridging this knowledge gap, the review offers insights that could refine perioperative management strategies and improve renal outcomes in cardiac surgery patients (Figure 1).

## Study Design

 This scoping review was carried out over an intensive 12-week period. Although the timeline was accelerated, methodological rigor and accuracy were consistently upheld. The review process was structured according to Arksey and O’Malley’s scoping review framework^16^ and aligned with the PRISMA-ScR reporting criteria.^9,17^ We implemented the five core phases described by Arksey and O’Malley^16,18^:

- Stage 1: Defining the study objectives and formulating the key research questions;
- Stage 2: Conducting a comprehensive search to locate relevant literature;
- Stage 3: Screening and selecting studies based on the established criteria;
- Stage 4: Systematically charting and organizing the extracted data;
- Stage 5: Integrating, synthesizing, and presenting the findings.

This scoping review was conducted between October and December 2024 and adhered to the methodological framework proposed by Arksey and O’Malley, with additional refinement using the PRISMA-ScR (Preferred Reporting Items for Systematic Reviews and Meta-Analyses extension for Scoping Reviews) checklist to enhance transparency and methodological rigor. A scoping review represents a systematic approach to mapping the extent, range, and nature of research evidence on a given topic, rather than establishing causal relationships or evaluating intervention effectiveness. This approach is particularly appropriate when the existing literature is heterogeneous or inconclusive, as is the case with CVP and its relationship to postoperative renal outcomes. Although CVP is routinely monitored in cardiac surgical practice to assess fluid status and cardiac function, its specific role in predicting AKI following CABG remains underexplored. Accordingly, this review sought to map and synthesize current evidence regarding the association between CVP and AKI in adult patients undergoing CABG — either isolated or combined with minor concurrent procedures. By identifying key patterns, methodological limitations, and knowledge gaps across existing studies, this scoping review aims to inform future research directions and support the development of evidence-based strategies to improve perioperative management and renal outcomes in CABG patients.^19,20^

 The scope of this review was established using the Population–Concept–Context (PCC) framework:

- Population: Adult patients undergoing cardiac surgery, including isolated CABG and CABG combined with valve procedures, while excluding isolated valve or aortic operations.
- Concept: CVP thresholds and the timing of their assessment (before, during, and after surgery) in relation to the onset of AKI.
- Context: Perioperative cardiac surgery settings encompassing intraoperative care and postoperative management in intensive care units.
- Outcome: Incidence and severity of AKI based on standardized diagnostic criteria such as KDIGO, RIFLE, or AKIN.
- Study Design: Observational studies (including prospective and retrospective cohorts) and randomized controlled trials (RCTs) were considered to provide comprehensive coverage of the existing evidence.

This design enabled mapping of evidence across study types, identifying the extent of current knowledge and highlighting the methodological limitations that influence interpretation of CVP–AKI associations.

###  Research Questions

 For this scoping review, the following research questions were formulated:

- What is the association between elevated CVP and development of AKI in adult patients undergoing cardiac surgery, with particular focus on CABG performed alone or in combination with valve procedures?
- Which perioperative CVP thresholds (measured preoperatively, intraoperatively, or postoperatively) are most strongly associated with the incidence and severity of AKI following cardiac surgery?
- How do the timing, duration, and magnitude of CVP elevation influence renal outcomes and recovery trajectories in this population?
- What implications do these findings have for integrating CVP monitoring into perioperative hemodynamic management protocols to prevent or mitigate CSA-A?

###  Search Strategy

 An independent preliminary search was conducted across major scholarly databases, including Scopus, Web of Science, PubMed, and MEDLINE. The MeSH headings and keywords used in the search strategy included: AKI (renal failure, renal insufficiency), cardiac surgery (heart surgery, cardiovascular surgical procedure, coronary bypass surgery, coronary artery surgery), and CVP. The incorporation of synonyms and related terms expanded the scope of the search. Boolean operators (AND, OR) and truncation symbols were used to combine and expand search terms appropriately. To maximize retrieval of relevant literature, the reference lists of all included studies were manually screened to identify additional eligible publications. Initial screening was based on titles and abstracts to exclude studies that did not meet the eligibility criteria. Any discrepancies in selection were resolved through discussion among the four reviewers, with final inclusion decisions made by consensus. Data were systematically extracted from all selected studies using a standardized form, capturing key elements such as authorship, study objectives, participant characteristics, interventions (where applicable), outcomes, and main findings. The search strategy focused on studies published between January 2016 and December 2024 to ensure relevance to contemporary clinical practice.

###  Study Selection

 The screening process was performed by pairs of authors who independently reviewed the titles, abstracts, and full texts of the retrieved studies. Study selection was guided by predetermined inclusion and exclusion criteria. A pre-designed data collection form was utilized, which was pilot-tested by two reviewers using four articles. No discrepancies were identified during the data extraction process.

###  Inclusion and Exclusion Criteria

 Studies were included if they fulfilled the predefined eligibility criteria outlined below:

- Population: Adult patients (aged ≥ 18 years) undergoing isolated CABG or CABG combined with minor concomitant procedures, excluding those involving primary valve replacement/repair or major aortic surgery.
- Outcome: Reporting of AKI incidence defined using standardized diagnostic criteria, primarily the Kidney Disease: Improving Global Outcomes (KDIGO) guidelines.
- Study design: Observational studies (prospective or retrospective cohorts) or randomized controlled trials published between January 2016 and December 2024.
- Language: Full-text articles published in English.

####  Exclusion Criteria Comprised

- Studies without a clearly specified AKI definition,
- Those lacking sufficient data on CVP measurements,
- Research conducted in pediatric populations or non-cardiac surgical settings,
- Narrative reviews, editorials, commentaries, and non-peer-reviewed publications.
- All identified articles were imported into EndNote version 9 for reference management, and duplicate records were systematically removed.

Two screening phases followed: initially, three independent reviewers evaluated titles and abstracts against the eligibility criteria. In the second phase, full texts were assessed for final inclusion. Any disagreements were resolved through discussion, with a fourth senior researcher making the final adjudication when consensus could not be reached.

###  Data Extraction and Synthesis

 All screening stages—title, abstract, and full-text—were conducted independently by two reviewers in duplicate, and data extraction was cross-verified by a second reviewer to ensure comprehensiveness and transparency. Any disagreements were resolved through discussion, and unresolved issues were adjudicated by a third senior reviewer. The workflow and reviewer assignments were managed using Covidence systematic review software, ensuring traceability and minimizing bias. Each included study was reviewed to capture the following data fields:

- Bibliographic information: authors, publication year, country, and study design.
- Study population: sample size, demographic characteristics, and type of cardiac surgery (isolated CABG or CABG with minor valve procedures).
- Clinical parameters: CVP measurement protocols (timing, duration, and threshold values), perioperative monitoring phase (pre-, intra-, or postoperative), and fluid management strategies.
- Outcome measures: definitions and diagnostic criteria for AKI, including KDIGO, RIFLE, or AKIN classifications, and reported clinical outcomes such as AKI incidence, severity, renal recovery, mortality, and ICU/hospital length of stay.

A structured Excel data extraction template was employed to maintain uniformity and minimize potential subjective bias. Extracted data were summarized in tabular format to facilitate cross-study comparison.

 In line with the methodological principles of scoping reviews, we did not conduct a formal quality assessment or evaluate the risk of bias. Rather, we employed a narrative and thematic synthesis, guided by the Arksey and O’Malley framework and the PRISMA-ScR reporting standards. The studies were grouped thematically according to (1) the phase of CVP monitoring (pre-, intra-, or postoperative), (2) the reported relationship between CVP elevation and AKI, and (3) the methodological diversity across studies. This structured synthesis enabled the identification of key evidence patterns and knowledge gaps, forming a robust foundation for future research recommendations.

## Results

**Figure 2:**
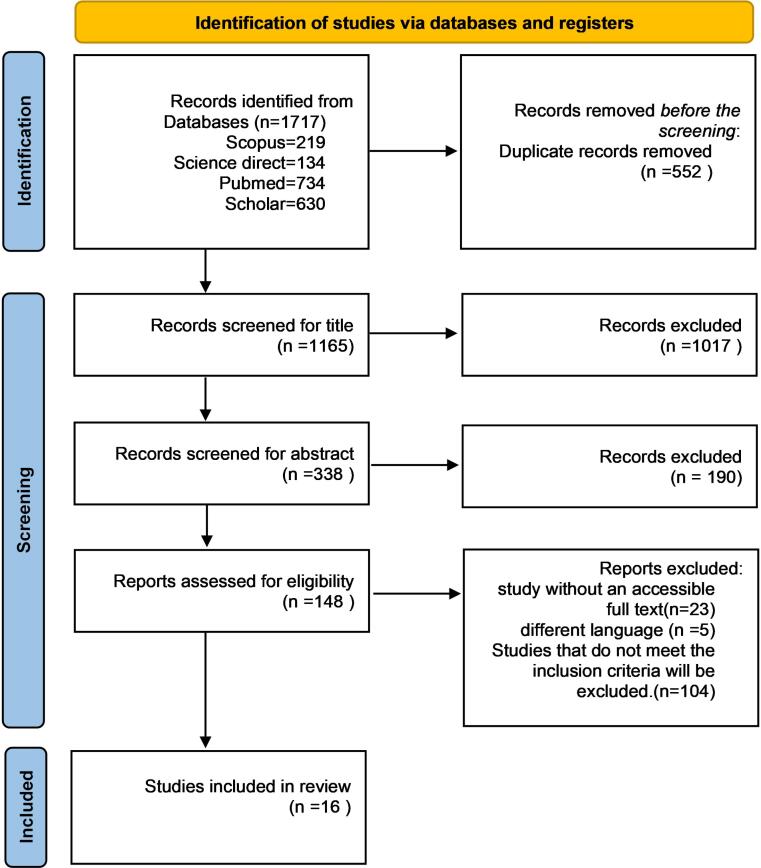
Flow Chart the Literature Search Strategy

 This scoping review study was conducted during the second half of 2024. The initial search identified 1,717 studies. After removing 552 duplicates, a total of 1,165 studies remained. Following title screening, 1,017 studies were excluded. The abstracts of the remaining 338 studies were reviewed. From the abstract review, 241 studies focused exclusively on postoperative AKI following CABG without addressing the role of CVP; 97 studies investigated the impact of CVP on patient mortality but did not discuss AKI outcomes. Consequently, 148 studies were selected for full-text review. Of these, 23 studies had inaccessible full texts, 3 studies addressed pediatric anesthesia, which was beyond the scope of this review, 5 studies were published in languages that did not meet the inclusion criteria, 42 studies discussed preoperative AKI exclusively, and 59 studies explored factors contributing to postoperative AKI but did not provide relevant results. Ultimately, 16 studies met the final inclusion criteria (Figure 2). These studies defined postoperative AKI using specific and standardized criteria; reported on CVP measurement time points and evaluated the association between CVP and AKI outcomes; and specifically focused on patients undergoing CABG surgery (Table 1).

**Table 1:** Summary of Studies Discussing the Relationship Between Central Venous Pressure and the Incidence of Renal Failure After CABG

**Year/** **Author**	**Country/center**	**Study Design**	**Sample size (n), population**	**CVP measurement (timing & threshold)**	**AKI criteria**	**Main findings**
2024, Demirjian et al^21^	USA	A Retrospective Cohort Study	60,424 cardiac surgery patients (CABG, valve, aorta(	Post-closure & ICU admission; continuous pulmonary artery catheter measurements	The primary outcome was the occurrence of moderate to severe AKI, defined as stage 2 or higher according to the modified KDIGO^a^ creatinine-based criteria, within a 14-day period following surgery.	Elevated CVP + low MAP → synergistic ↑AKI risk (*P* = 0.009)
2024. Wang et al^5^	China	A Retrospective Cohort Study	2,048 cardiac surgeries with CPB	Intra-CPB; high CVP ≥ 6.5 mm Hg	KDIGO	High CVP ( ≥ 6.5 mm Hg) independently predicted AKI (*P* = 0.017)
2023, Dang et al^22^	USA	A retrospective cohort study	23 cardiac surgery adults	Pre-CPB & ICU; dynamic	KDIGO	↑CVP → ↓effective renal perfusion pressure → ↑AKI (*P* < 0.05)
2022, Alhulaibi et al^23^	Saudi Arabia	A Retrospective Cohort Study	329 cardiac surgery with CPB	Post-op CVP via PAC	KDIGO	AKI group CVP = 11.5 ± 2.7 > controls (10.4 ± 2.5 mm Hg) (*P* = 0.003)
2022, Tohi et al^24^	Japan	A prospective observational study	64 CPB surgeries	Intra-op continuous PAC	KDIGO	Dynamic ↑CVP strongly correlated with AKI (*P* < 0.0001)
2021, Neuman et al^25^	Australia	An observational cohort study	221 cardiac surgeries	Post-op ICU	KDIGO	CVP ≥ 9 mm Hg→ ↑AKI risk (*P* = 0.01)
2021, Lopez et al^26^	USA	An observational cohort study	425 elective CABG/valve/aorta	Intra-op continuous CVP (excluding CPB)	KDIGO	↑CVP (60 mm Hg/min) → 6–30% higher AKI risk (*P* = 0.008)
2021, Kotani et al^27^	Japan	A multicenter retrospective cohort study	746 CABG/valve	Pre- & post-op CVP (1h intervals, 24 h)	KDIGO	↑time-weighted CVP → AKI progression OR 1.12 (*P* = 0.0013)
2020, Chen et al^14^	China	A Retrospective Cohort Study	5,127 CABG/valve	Pre/post-CPB CVP (12–20 mm Hg thresholds)	KDIGO	Venous congestion > hypotension in AKI prediction
2020, Farghaly et al^28^	Egypt	A Prospective Observational Study	100Post-cardiac vasopressor patients post operative	Pre- & post-op CVP (24 h mean)	KDIGO	↑CVP → 50% AKI incidence (*P* < 0.001)
2019, Raymond Hu el^29^	Australia	A retrospective, observational cohort study	664 CPB	Baseline & post-induction CVP	AKI was defined by a > 50% rise in serum creatinine from baseline within 7 postoperative days, per RIFLE^b^ criteria.	Baseline CVP → independent AKI predictor, along with factors like age, preoperative creatinine, and LV dysfunction (*P* < 0.05).
2019, Jin et al^30^	China	A retrospective analysis	300 post-cardiac surgery	Post-op 24 h CVP	KDIGO	Patients with AKI exhibited significantly higher CVP values, both at peak (10.7 ± 3.5 vs. 9.8 ± 2.7 mm Hg) and nadir (5.0 ± 2.2 vs. 4.5 ± 1.8 mm Hg), compared to those without AKI (*P* < 0.05).
2019, Che et al^31^	China	Observational study	2,552 mixed cardiac	Post-op CVP < 4.4 or > 10.3 cmH₂O	KDIGO	CVP of less than 4.4 mm Hg or greater than 10.3 mm Hg in the postoperative period→ ↑AKI risk (*P* < 0.001).
2018, Jiang et al^32^	China	A propensity score-matched case-control study	1,773 CABG/valve	Postoperative CVP was extracted from the cardiac surgery database of Zhongshan Hospital	KDIGO	CVP > 7.35 mm Hg at ICU admission independently predicted AKI-RRT after cardiac surgery (OR = 1.6 per 1 mm Hg increase; *P* < 0.05).
2018, Beaubien-Souligny et al^33^	Canada	A prospective observational cohort study	145 CABG/CPB	Post-op CVP continuous	KDIGO	↑CVP → ↑AKI risk (with a hazard ratio 1.04 per mm Hg) (*P* = 0.02).
2017, Jiang et al^34^	China	A retrospective cohort study	1,587 CABG/valve	Post-op ICU CVP	KDIGO	AKI group )8.0 ± 2.1 mm Hg vs. 6.2 ± 2.0 mm Hg (*P* < 0.01)

^a^Kidney Disease: Improving Global Outcomes (KIDGO) defined as: (1) ΔCr ≥ 0.3 mg/dL within 48 h, (2) Cr ≥ 1.5 × baseline within 7 days, or (3) urine output < 0.5 mL/kg/h for > 6 h ^5^. ^b^The RIFLE classification, proposed by Bellomo et al, defines the first stage of AKI as a decrease of more than 25% in the estimated glomerular filtration rate (GFR).^23,35^

 Across the studies, associations between elevated CVP and AKI were generally positive but varied in magnitude and threshold (ranging 6.5–12 mm Hg intraoperatively, > 6.6–10.3 mm Hg postoperatively). Variability in AKI definitions (KDIGO, RIFLE, AKIN) and timing of CVP assessment contributes to the heterogeneity. The included studies primarily involved adult patients undergoing CABG, either isolated or combined with minor concomitant procedures, with CVP measured intraoperatively or postoperatively.

 To ensure transparency and reproducibility, only peer-reviewed articles meeting predefined inclusion criteria were considered. All study selection decisions were documented, and inter-rater agreement during screening and full-text review was high (Cohen’s kappa > 0.80), ensuring consistency across reviewers. No formal risk-of-bias or quality assessment was conducted, in line with scoping review methodology.

 The results indicate that elevated CVP is frequently associated with postoperative AKI. Studies consistently demonstrated that higher CVP, particularly when combined with other hemodynamic factors such as low MAP, increased the likelihood of AKI. However, differences in CVP measurement protocols, threshold values, and AKI classification systems highlight the need for standardized definitions and measurement practices in future research. These findings underscore the potential role of CVP monitoring in perioperative care and provide a foundation for future prospective studies aimed at developing CVP-guided strategies to mitigate postoperative renal complications in CABG patients.

###  Summary of Evidence

 This scoping review synthesizes evidence regarding the potential predictive role of CVP for AKI following CABG. The included studies provide valuable insight into the relationship between CVP and AKI incidence, examining both measurement timing and the magnitude of association. However, across the studies, the findings are heterogeneous with respect to CVP thresholds, measurement timing, and AKI definitions. CVP thresholds associated with increased AKI risk varied widely (6.5–14 mm Hg intraoperatively, 6.6–12 mm Hg postoperatively), and some studies reported weak or non-significant associations (Table 1).^5,14,25,31^ Most studies used KDIGO guidelines, though RIFLE and other non-standard definitions were also applied.^29^

###  Principal Findings

 Elevated CVP consistently emerged as an independent predictor of postoperative AKI across diverse patient populations and study designs. CVP thresholds associated with increased AKI risk varied, ranging from 6.5 mm Hg to ≥ 12 mm Hg, with postoperative CVP measurements showing the strongest association. According to the studies referenced, elevated CVP was found to significantly contribute to an increased risk of AKI, which in turn was associated with higher mortality rates.^5,14,25,30,31,34^

 Although all included studies demonstrated a significant relationship between elevated CVP and the occurrence of AKI, there was substantial variability in the CVP cutoff values used, the timing of measurements (intraoperative versus postoperative), and the magnitude of the reported associations. This heterogeneity suggests that CVP is not a universally applicable predictor and cautions against broad clinical recommendations in the absence of standardized protocols.

 Venous congestion may contribute to renal dysfunction through several mechanisms:

1. Reduced renal perfusion pressure: Elevated CVP decreases the renal arteriovenous pressure gradient, impairing glomerular filtration and renal blood flow.^26^
2. Intrarenal congestion: Increased intrarenal pressure from elevated CVP directly contributes to structural and functional renal impairment.^7,26^
3. Neutrophil accumulation: High CVP can impede renal blood flow, leading to neutrophil accumulation in peritubular capillaries and upregulation of inflammatory signals, potentially causing kidney damage.^5^

Moreover, the review highlighted a synergistic relationship between elevated CVP and low MAP, wherein the combination significantly increased AKI risk. This finding emphasizes the need for balanced hemodynamic management, targeting both arterial and venous pressures, to mitigate renal complications.^21^

## Clinical Implications

 While CVP monitoring may provide useful information for perioperative risk assessment in CABG patients, the current evidence does not support a uniform CVP threshold or routine use to guide interventions in isolation. Interventions such as fluid management, inotropes, or diuretics should be individualized based on patient-specific risk factors, intraoperative events, and postoperative hemodynamic status.^5,12,14,21,26,36^

## Study Strengths and Limitations

 We excluded studies published prior to 2016 to reduce the risk of temporal bias. This review is strengthened by the use of a systematic approach, compliance with PRISMA-ScR reporting standards, and the incorporation of heterogeneous study populations, all of which improve the overall applicability of the results. However, the review is limited by its reliance on observational data, heterogeneity in CVP measurement techniques, timing, and thresholds, variability in AKI definitions, and differences in patient populations and surgical practices. Additionally, only studies published in English were included, introducing potential language bias.

 Despite the consistency of findings, significant heterogeneity complicates direct comparison across studies. Some studies utilized intraoperative CVP monitoring^37,38^, while others focused on postoperative values,^25,30^ leading to variability in reported associations with AKI. These studies reported various CVP thresholds associated with an increased risk of AKI; however, they did not establish a precise or definitive CVP range. Furthermore, most studies rely on retrospective cohort designs, which limit causal inference.

## Future Directions

 Several avenues for future research emerge from this review:

- Standardization of CVP measurement and reporting protocols to reduce heterogeneity.
- Investigation of optimal CVP thresholds predictive of AKI in multicenter prospective studies.
- Integration of CVP into broader risk models, including MAP, cardiac output, and fluid balance.
- Intervention studies evaluating CVP-guided perioperative strategies, such as targeted fluid or diuretic

## Conclusion

 This scoping review maps and synthesizes current evidence on the association between CVP and AKI following cardiac surgery. Across the included studies, elevated CVP was generally associated with a higher risk of postoperative AKI, although thresholds, measurement timing, and AKI definitions varied.These findings highlight the potential role of CVP monitoring in perioperative hemodynamic management but do not establish causality due to observational study designs and methodological heterogeneity. Prospective, multicenter studies with standardized CVP protocols are needed to confirm these associations, identify optimal thresholds, and evaluate CVP-guided interventions to improve renal outcomes in CABG patients.
